# The Effects of Different Durations of Night-Time Supplementary Lighting on the Growth, Yield, Quality and Economic Returns of Tomato

**DOI:** 10.3390/plants13111516

**Published:** 2024-05-31

**Authors:** Hongjun Yu, Peng Liu, Jingcheng Xu, Tanyu Wang, Tao Lu, Jie Gao, Qiang Li, Weijie Jiang

**Affiliations:** 1 Institute of Vegetables and Flowers, Chinese Academy of Agricultural Sciences, Beijing 100081, China; yuhongjun@caas.cn (H.Y.); saaslp2023@163.com (P.L.); xujingcheng94@163.com (J.X.); tanyuwang1989@163.com (T.W.); lutao@caas.cn (T.L.); 2 Taizhou Academy of Agricultural Sciences, Taizhou 318014, China; 3 College of Horticulture, Xinjiang Agricultural University, Urumqi 830052, China; ofc111@163.com

**Keywords:** tomato, night-time supplemental light, yield, signal, economic returns

## Abstract

To achieve higher economic returns, we employ inexpensive valley electricity for night-time supplementary lighting (NSL) of tomato plants, investigating the effects of various durations of NSL on the growth, yield, and quality of tomato. Tomato plants were treated with supplementary light for a period of 0 h, 3 h, 4 h, and 5 h during the autumn–winter season. The findings revealed superior growth and yield of tomato plants exposed to 3 h, 4 h, and 5 h of NSL compared to their untreated counterparts. Notably, providing lighting for 3 h demonstrated greater yields per plant and per trough than 5 h exposure. To investigate if a reduced duration of NSL would display similar effects on the growth and yield of tomato plants, tomato plants received supplementary light for 0 h, 1 h, 2 h, and 3 h at night during the early spring season. Compared to the control group, the stem diameter, chlorophyll content, photosynthesis rate, and yield of tomatoes significantly increased upon supplementation with lighting. Furthermore, the input–output ratios of 1 h, 2 h, and 3 h NSL were calculated as 1:10.11, 1:4.38, and 1:3.92, respectively. Nonetheless, there was no detectable difference in yield between the 1 h, 2 h, and 3 h NSL groups. These findings imply that supplemental LED lighting at night affects tomato growth in the form of light signals. Night-time supplemental lighting duration of 1 h is beneficial to plant growth and yield, and its input–output ratio is the lowest, which is an appropriate NSL mode for tomato cultivation.

## 1. Introduction

In light of a recent investigation, solar greenhouses have become the most important facilities in protected vegetable production in northern China. Light deficiency is one of the critical environmental limiting factors affecting crop yield for the greenhouse vegetable production [1]. Light is a key environmental variable, whose effects on plant growth and development are mainly reflected in three aspects, including light intensity [2], light quality [3,4] and illumination time [5], which can regulate plant morphogenesis, photosynthesis [6], material metabolism and yield [7,8]. Poor irradiance is one of the major limiting factors for plant growth and development, especially for vegetative growth. Some papers have described that plants under low-light conditions suffer from improper metabolism and senescence acceleration [9,10]. Low solar radiation is harmful to the photosynthesis of rice, which hinders the accumulation and distribution of dry matter, resulting in a lower yield and poor quality of rice [11]. Artificial regulation and optimization of light conditions can improve the photosynthetic characteristics [12], promote crop growth [13], increase the yield [14], enhance the quality, and effectively delay the organ senescence of plants [15]. Some researchers have verified that light supplementation by LED can enhance the photosynthetic capacity of cucumber [16], and at bloom time, supplementary illumination can markedly promote the growth of cucumber fruits [17].

In recent years, LED supplementary lighting technology has been commonly used in protected agricultural production. Numerous studies have confirmed that LED lighting supplementation can be beneficial to crop growth and development, increase yield and improve quality [15,18]. Previously, several meaningful studies about the effects of light intensity and light quality on the performance of different plants were conducted [19,20], but there is little research available to elucidate the effects of NSL duration on tomato growth, especially studies on the effects of NSL duration on the whole growth cycle of tomato and the corresponding economic returns.

To enhance the energy resource utilization efficiency and incentivize electricity consumers to allocate electrical power consumption rationally, utility firms segment 24 h per day into peak (8:00–22:00) and off-peak (22:00–8:00) intervals, offering varying electricity rates during these respective timescales. During our experimental setup, we circumvent the most heavily utilized interval of electric power consumption (i.e., the ‘peak’ period), instead opting to initiate illumination once the cost of electricity plummets (i.e., the ‘valley’ period). The primary aim of this study is to investigate the influence of night-time supplementary light (NSL) on tomato development and fruitfulness throughout various developmental phases, as well as assess its impact on tomato yield and potential monetary rewards. No conclusive association was observed between the length of additional lighting and tomato yield, despite previous findings from daylight studies suggesting that such treatment’s advantages escalated alongside extended exposure durations [21,22]. Consequently, we aimed to verify if the enhancement in crop growth and output induced by nocturnal supplementary illumination correlates with lighting duration. As a means to delve deeper into this phenomenon, we reduced the elongating photoperiod, adding an experiment encompassing supplementary lighting durations of 1 h, 2 h, and 3 h. Our investigations served to thoroughly evaluate the effects of NSL upon tomato development, identify the optimal lighting duration, and establish a solid foundation for developing a low-cost yet highly advantageous nocturnal supplementary lighting technique capable of widespread implementation.

## 2. Materials and Methods

### 2.1. Experimental Materials

The tomato variety DER0899 was selected as the plant material in this experiment. Tomato seeds were successfully germinated and sown in 32-plug trays on 18 July 2016 and 10 January 2017, respectively. Seedlings reaching the four true-leaf stage (10 August 2016 and 22 February 2017, respectively) were transplanted into cultivation trough (7 m long × 0.35 m wide × 0.15 m deep) and each trough had 39 tomato seedlings. The cultivation substrate was cocopeat, provided by Beijing Yinong Agricultural Technology Company, Beijing, China. The organic fertilizer used in this trial was produced by Qian’an Fusheng Animal Husbandry Technology Company, Tangshan, Hebei, China. This organic fertilizer was blended with coconut coir at a rate of 55 kg:1 m^3^ (*w*/*v*). The drip irrigation system used an integrated precision automatic control system provided by Beijing Zigvine Technology Company, Beijing, China. With soil humidity ≤ 40% and light intensity ≥ 450 μmol·m^−2^·s^−1^, the drip irrigation system started and ran for four minutes. The planting density was 4 plants per square meter. The formula of the nutrient solution used in the experiment is the special Yamazaki tomato formula (Table 1) [23].

LED tubes (97 cm × 10 cm) used as supplemental lighting sources were obtained from Chongqing Xinglian Yunke Technology Development Co., Ltd, Chongqing, China. The total power of each LED tube was 70W, and the red–blue light ratio was 2:1, producing an estimated photosynthetically active photon flux density (PPFD) of approximately 88 μmol·m^−2^·s^−1^ when measured at a distance of 10cm from the LED module. For each cultivation trough, four LED tubes were installed vertically above the plant canopy at a height of around 50 cm, with the distances between the LED tubes and the plant canopy adjusted based on the plant’s growth and kept constant during the entire experiment. The measured light intensity reaching the plant canopy was approximately 30 μmol·m^−2^·s^−1^.

### 2.2. Experimental Design

The NSL trial of the autumn–winter season (AW) tomato was conducted in the solar greenhouse of the Xiaotangshan special vegetable base. The experiment consisted of 4 treatments, including control (without lighting supplementation), supplemental lighting for 0, 3 h (23:00~02:00), 4 h (23:00~03:00), 5 h (23:00~04:00). To tomato plants with six true leaves, supplemental lighting at night was applied from 17 August 2016 to 30 October 2016. The experiment consisted of three replicates and the total number of the four treatments was 468, and each cultivation trough was used as a replication. The average sunlight exposure time in the greenhouse was 8 h per day, with an average light intensity of 250 μmol·m^−2^·s^−1^. The temperature inside the greenhouse was 23–26 °C and 10–12 °C during the day and night, respectively.

In order to further explore the effect of the duration of NSL on tomato production, the NSL trial of the early spring (ES) season tomato was conducted in the solar greenhouse of the Xiaotangshan special vegetable base. The experiment consisted of 4 treatments, including control (without lighting supplementation), supplemental lighting for 1 h (23:00~00:00), 2 h (23:00~01:00), 3 h (23:00~02:00). To tomato plants with six true leaves, supplemental lighting at night was applied from 28 February 2017 to 14 May 2017. The experiment consisted of three replicates and the total number of the four treatments was 468, and each trough was used as a replication. The average sunlight exposure time in the greenhouse was 10 h per day, with an average light intensity of 300 μmol·m^−2^·s^−1^. The temperature inside the greenhouse was 26–32 °C and 15–18 °C during the day and night, respectively.

Black clothes were used for blocking the light from adjacent troughs of other treatments at night, and they were removed during the day. The drip irrigation system, a water- and fertilizer-integrated precision automatic control system that could automatically irrigate based on substrate humidity and light intensity, was employed in this trial. After completion of these trials, the values of each electric meter were recorded for use in calculating the energy consumption per tank.

### 2.3. Measurements and Methods

#### 2.3.1. Plant Growth

On days 15, 30, 45, 60, and 75 following the illumination treatment (DAT), plant height, stem diameter (measured using a scale with the fifth leaf beneath the apex closed), and chlorophyll content were measured from 18 randomly selected plants from each group during each observation period using ruler, electronic vernier caliper, and SPAD-502 Plus chlorophyll meter, respectively.

#### 2.3.2. Leaf Net Photosynthesis Rate

At 15, 45, and 75 days post initiation of supplementary lighting, the net photosynthesis rate for three independently growing tomato plants per experimental replicate was assessed using a portable LI-6400 photosynthesis system (LI-6400XT, Li-COR, Inc., Lincoln, NE, USA) as described previously [24]. The parameters used were as follows: photosynthetic photon flux density, 1000 μmol·m^−2^·s^−1^; and air flow rate inside the sample chamber, 400 mol·s^−1^.

#### 2.3.3. Fruit Yield

The yield per trough for each treatment was calculated by the cumulative yield throughout the harvest time, yield per plant, and single-fruit weight of 18 selected tomato plants weighed by an electronic balance.

#### 2.3.4. Fruit Quality

Ripe tomato fruits with uniform maturity were randomly selected from each treatment to measure the nutritional quality parameters, including titratable acid content, soluble sugar content, Vitamin C content and soluble solids’ content, determined by titration with sodium hydroxide, anthrone-sulfuric acid colorimetry, spectrophotometer colorimetry and portable Abbe refractometer, respectively.

### 2.4. Statistical Analysis

All data were analyzed using Excel 2003 and SPSS 17.0 software, and the statistical significance of the differences between treatments was determined by Duncan’s multiple range test (*p* < 0.05). Different letters indicate significant differences.

## 3. Results

### 3.1. Effects of NSL Duration on Plant Height of Tomato

**Figure 1:**
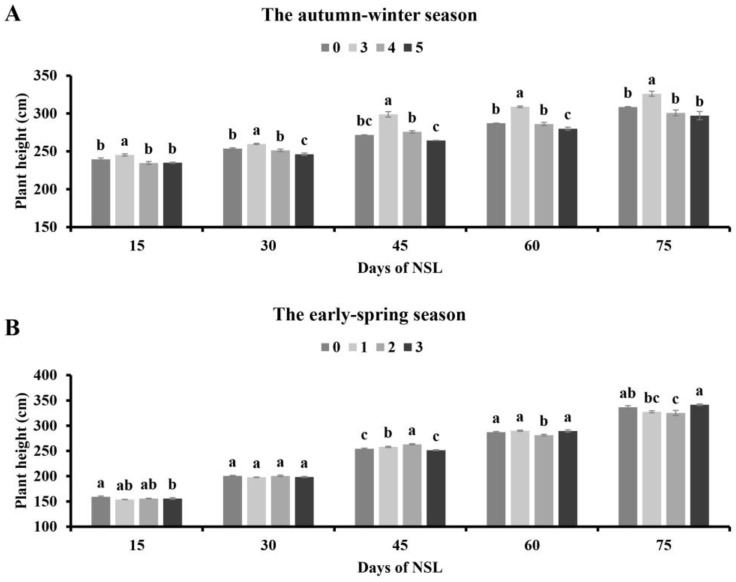
Effects of NSL duration on plant height of tomato in the autumn–winter season (**A**) and the early spring season (**B**). Different letters indicate statistically significant differences (*p* < 0.05).

In the autumn–winter season, the plant height of tomatoes cultivated under 3 h NSL was strikingly higher than that of other treatments. And there was no significant difference between the plant height of tomatoes cultivated under 4 h and that of the control. However, compared with the control, the plant height of tomatoes under the 5 h supplementary lighting treatment decreased markedly 30 and 60 days after the lighting treatments (DAT), and no difference was observed at other times (Figure 1A).

In the early spring season, the tomato plants to which artificial night-time lighting for 1 h was applied were shorter than the plants without lighting supplementation after 15 days. However, there was no difference among the four treatments 30 DAT. Additionally, the tomatoes receiving the 2 h supplemental lighting treatment were obviously taller than those of other groups on 45 DAT, but the plant height of lighting treatments increased from 60 to 75 DAT, and the plant height of the 2 h night-time lighting supplementation group was markedly lower than that of the other treatments. Moreover, there was no difference in tomato plant height between those subjected to lighting for under 3 h and those without lighting (Figure 1B).

### 3.2. Effects of NSL Duration on Stem Diameter of Tomato

**Figure 2:**
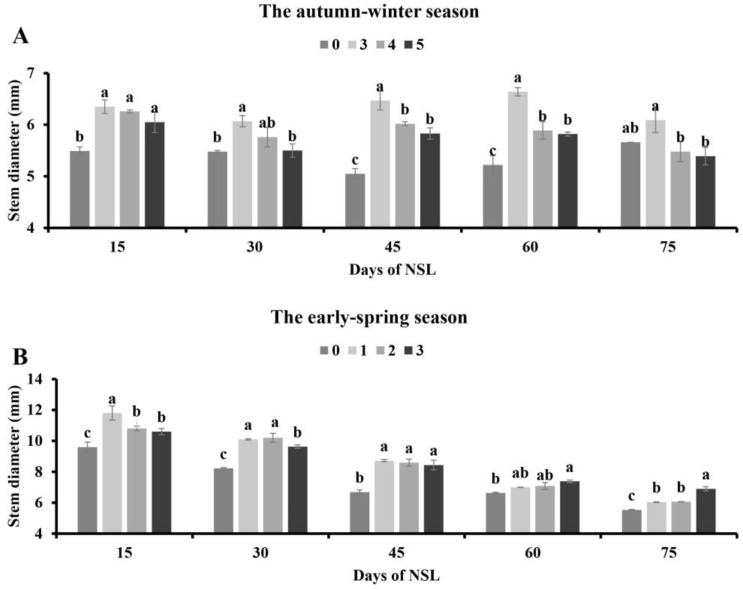
Effects of NSL duration on stem diameter of tomato plants in the autumn–winter season (**A**) and the early spring season (**B**). Different letters indicate statistically significant differences (*p* < 0.05).

In the autumn–winter season, compared to the control, the exposure of tomato plants to supplementary lighting contributed to a marked increase in stem diameter from 15 to 60 DAT. The tomato stem diameter of the 3 h lighting treatment was remarkably increased, except on 75 DAT. And the tomato stem diameter of the 4 h and 5 h lighting treatments was increased significantly except on 30 and 75 DAT. Among different lighting treatments, the stem diameter of tomato was similar 15 DAT, but the group in which supplemental lighting for 3 h was used had a thicker stem diameter than that with 5 h lighting 30 DAT. In addition, the group of supplemental lighting for 3 h showed better behavior than the 4 h and 5 h groups regarding the tomato stem diameter from 45 to 75 DAT (Figure 2A).

In the early spring season, compared to the control, the three supplementary illumination treatments could remarkably increase the stem diameter, except on 60 DAT. The tomato stem diameter of the 3 h of NSL group increased strikingly 60 DAT compared to that of the control. Among the various durations of the NSL treatments, exposing tomato plants to 1 h NSL showed a better result than 2 h and 3 h on stem diameter 15 DAT. Tomato plants treated with supplemental lighting for 1 h and 2 h had a thicker stem diameter than 3 h 30 DAT. No significant difference in tomato stem diameter was noted among those lighting supplementation treatments on 45 and 60 DAT. The stem index was remarkably higher by the addition of lighting for 3 h than 1 h and 2 h, but a non-significant difference was detected between the treatments with lighting for 1 h and 2 h at 75 DAT (Figure 2B).

### 3.3. Effects of NSL Duration on Chlorophyll Content

**Figure 3:**
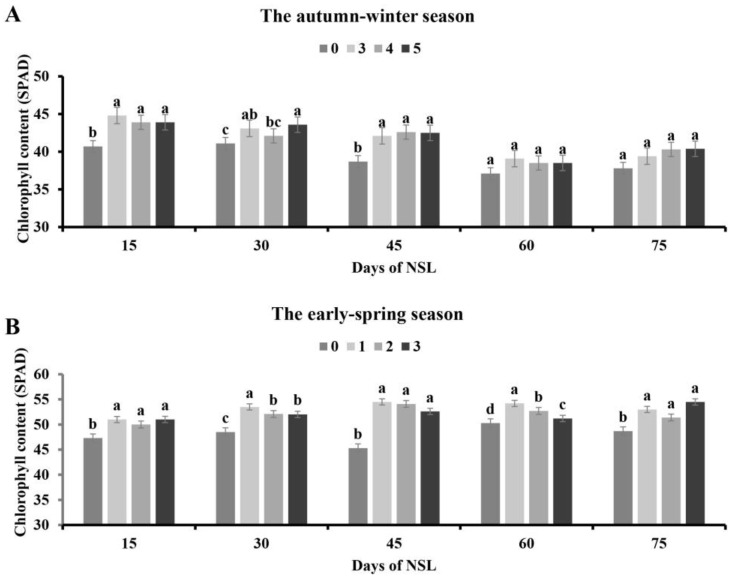
Effects of NSL duration on the chlorophyll content of tomato leaves in the autumn–winter season (**A**) and the early spring season (**B**). Different letters indicate statistically significant differences (*p* < 0.05).

In the autumn–winter season, compared to the control, all three night-time supplemental lighting treatments could obviously enhance the chlorophyll content presented in tomato leaves on 15 and 45 DAT, but there was no significant difference among the three lighting groups. A total of 30 DAT, the chlorophyll content of tomato plants receiving 3 h and 5 h night-time lighting supplementation tended to be markedly higher than that of plants without supplementing lighting. And the 5 h supplementary illumination treatment led to significantly higher chlorophyll content than the 3 h lighting treatment, while no difference was observed between the 3 h and 4 h lighting treatments. The chlorophyll content was similar in the absence or presence of supplemental illumination 60 DAT (Figure 3A).

In the early spring season, the chlorophyll content of the supplemental lighting groups was significantly increased compared to that of the control from 15 to 75 DAT. Groups utilizing night-time lighting supplementation had the same effect on the SPAD value. The chlorophyll content of tomato plants cultured under 1 h night-time lighting tended to be markedly higher than that of the other two lighting treatments, and there was no remarkable difference between the other two lighting groups on 30 DAT. A total of 60 DAT, tomato plants exposed to 1 h lighting supplementation contained a markedly higher chlorophyll content relative to the other two supplemental lighting treatments, and the 3 h lighting group had a lower chlorophyll content than the 2 h lighting group (Figure 3B).

### 3.4. Effects of NSL Duration on Net Photosynthetic Rate

**Figure 4:**
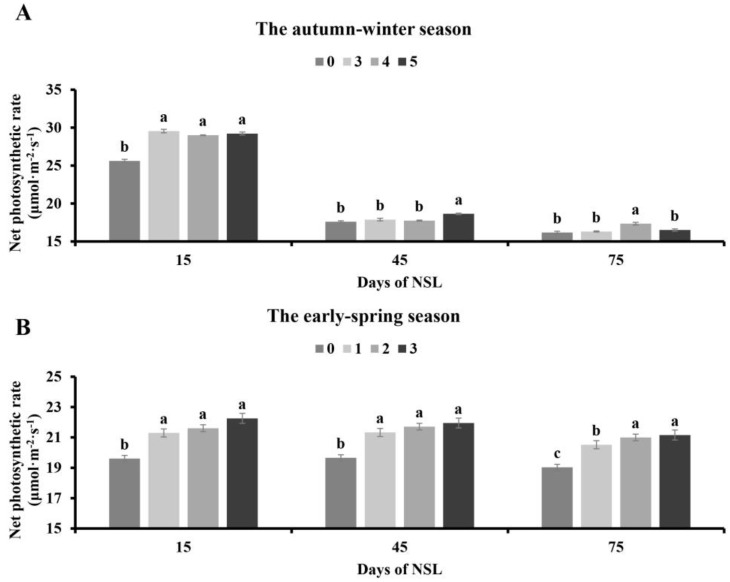
Effects of NSL duration on net photosynthetic rate of the autumn–winter season tomato (**A**) and the early spring season tomato (**B**). Different letters indicate statistically significant differences (*p* < 0.05).

In the autumn–winter season, the net photosynthetic rate of the supplemental lighting groups was significantly enhanced compared to that of the control while no obvious difference was noted among the lighting supplementation treatments on 15 DAT. The net photosynthetic rate of the tomato plants with 5 h lighting was markedly higher than that of the other groups 45 DAT, while the other three groups had a consistent effect on the photosynthetic rate. On 75 DAT, the net photosynthetic rate of tomato plants grown under 4 h night-time lighting supplementation tended to be obviously higher than the other treatments’, but there was no remarkably different treatment effect between the control and the other two lighting groups (Figure 4A).

In the early spring season, it can be observed that compared with the control, the net photosynthetic rate enhanced obviously in all different lighting supplementation treatment groups. No significant difference in the net photosynthetic rate was noted between the three lighting treatments on 15 and 45 DAT. Supplementing lighting for 2 h and 3 h had the same influence of photosynthetic capability, and the 1 h lighting treatment had a better effect than the other two lighting treatments on the photosynthetic parameter (Figure 4B).

### 3.5. Effects of NSL Duration on Tomato Yield and Input–Output Ratio

In the autumn–winter season, compared with the no additional lighting group, the single-fruit weight, yield per plant and yield per trough of the lighting supplementation groups increased significantly; however, those night-time supplemental lighting treatments exerted an equivalent influence on single-fruit weight. Supplementing lighting for 3 h showed better performances than 5 h on yield per plant and yield per trough. Among the three lighting groups, the 3 h supplemental lighting group had the least electricity input, accompanied by the highest increased yield per trough and the biggest input–output ratio 1:6.37 (Table 2).

In the early spring season, the treatment of 3 h NSL led to significantly higher single-fruit weight than that of the control, while the other two supplemental lighting treatments matched up with the control on this index. Among the three lighting groups, no marked difference was found in single-fruit weight. The yield per plant and the yield per trough of all lighting addition treatments were significantly higher than those of the control. Although the electricity consumption was different among the three lighting supplementation groups, there was no significant difference in yield per plant and yield per trough. In summary, supplementing lighting for 1 h had the biggest input–output ratio, 1:10.11 (Table 2).

The input of electricity charge was CNY 0.3 per kWh, and the output of tomato fruit was CNY 4 per kilogram. The one-off input of LED tubes was not included in this table. Different letters indicate statistically significant differences (*p* < 0.05).

### 3.6. Effects of NSL Duration on Fruit Quality of Tomato

In the autumn–winter season, the concentration of Vitamin C and soluble solids in tomato fruits under night-time supplemental LED lighting for 3 h was significantly greater than that of the control, while titratable acidity was markedly lower than that of the control. The soluble sugar content of tomato fruits irradiated with 3 h supplementary illumination seemed to be higher than that of plants subjected to darkness at night but the difference was not significant between the two groups. Compared with the control, the content of titratable acid and Vitamin C in tomato fruits exposed to 4 h night-time lighting supplementation was markedly higher, and the content of soluble sugar and soluble solids was also higher than that of the control but the difference did not reach a significant level. The tomato fruits of the 5 h night-time supplementary illumination group stored remarkably more titratable acid, soluble sugar and soluble solids than those of the control, while no remarkable difference in Vitamin C content was found between them. The 3 h supplemental lighting application group had the best performance on the acid–sugar ratio and total soluble solids–acid ratio among the four treatments (Table 3).

In the early spring season, the 1 h lighting treatment improved the content of soluble solids compared to the control and the 3 h lighting group; in addition, titratable acidity was significantly lower than that of the control, and the content of Vitamin C and soluble sugar was found to be insignificant compared with that of the control. The content of soluble sugar, Vitamin C and soluble solids accumulated in tomato fruits exposed to 2 h supplementary illumination was markedly modified compared tot to that of the other treatments. The content of titratable acid of tomato fruits exposed to 2 h night-time lighting supplementation was significantly higher than that of the 1 h and 3 h lighting treatments but was insignificant compared with that of the control. The tomato fruits of the 3 h night-time supplementary illumination group contained considerably less titratable acid than those of the control but had an equal level of soluble sugar, Vitamin C and soluble solids compared with the control. The control had a lower acid–sugar ratio and total soluble solids–acid ratio than all supplementary illumination treatments, indicating that supplemental lighting at night had a positive effect on the quality improvement of tomato fruits (Table 3).

## 4. Discussion

### 4.1. NSL Affects Tomato Production in the Form of Light Signals

With the wide application of LED lights, it is possible to supplement lighting in plant factories and facilities [25]. Recently, numerous studies have examined the effects of various factors related to supplementary lighting duration, quality [26], and intensity [27] on plant growth during the day. However, there is only a limited number of studies examining the impact of nocturnal supplementary lighting on plant development. In our study, we employed night-time supplementary lighting technology to disrupt the dark cycle of plants. Additionally, the intensity of supplementary lighting used in this experiment at night was 30 μmol·m^−2^·s^−1^, which did not supply sufficient energy for photosynthesis in tomatoes. Notably, our findings indicate that nocturnal supplementary lighting can substantially enhance the photosynthetic rate in the daytime, yield, and quality of tomatoes. Thus, we hypothesize that nocturnal supplementary lighting may serve as a type of light signal promoting plant growth.

Nocturnal supplementary lighting disrupts the circadian rhythms of plants, which can influence multiple aspects of plant physiology [28,29]. Additionally, Velez-Ramirez et al. found that the expression of CAB-13 (III light harvesting chlorophyll a/b binding protein 13) is regulated by the circadian rhythm, and this protein can regulate the effects of photosystem I and II light harvesting, which is an important factor affecting plant photosynthesis [30]. The results of our experiment show that supplemental lighting at night can improve the photosynthetic capacity during the whole production period of tomato in the early spring season and the early stage of tomato in the autumn–winter season (Figure 4), and that is consistent with the report by Tewolde et al. [31]. The chlorophyll content and distribution in plant leaf tissue also affects photosynthetic capacity. It has been reported that supplemental lighting at night increases the chlorophyll content in the middle and lower canopy of tomato [31]. This experiment also found that supplemental lighting at night increased the chlorophyll content of tomato (Figure 3). The increase in chlorophyll content may be one of the reasons why supplemental lighting at night promotes photosynthesis during the day.

Assimilate partitioning plays a crucial role in plant growth and morphological development [32]. Light qualities and photoperiods affect how assimilates distribute within plants [33,34]. Whether night-time supplementary lighting, acting as light signals, regulates the distribution of assimilates remains to be further investigated. These findings offer novel insights and approaches for regulating environmental light in plant factories and facilities.

### 4.2. The Duration of Night-Time Supplemental LED Has No Effect on Tomato Yield

In the present research, the findings of the night-time lighting supplementation experiment in autumn suggest that supplementing lighting for 3 h, 4 h and 5 h at night could promote the growth of tomato plants at the earlier growth stage and significantly increase the yield of tomato. However, supplementing lighting for 3 h showed better performances than 5 h on the yield per plant and yield per trough. To further refine the duration and verify our hypothesis that improved plant growth and yield through night-time supplementation might not necessarily correlate with the length of NSL, we reduced the durations from 3 h, 4 h, and 5 h to 1 h, 2 h, and 3 h during the early spring test, respectively. Nonetheless, there was no significant difference in single-fruit weight, yield per plant or yield per trough among the three lighting groups in the early spring season.

Interestingly, we discovered that extended exposure to night-time supplemental lighting does not guarantee an increased tomato yield. Under these conditions, the maximum yield and the highest input–output ratio were achieved when tomato plants received supplementary lighting for 1 h at night. This is inconsistent with many previous reports in the literature [35]. The rationale behind this phenomenon lies in the fact that the night-time supplementary lighting employed in this study functions as light signals to disrupt the tomato’s dark cycle. Moreover, excessive supplementary lighting can lead to both energy wastage and detrimental effects on plants [36,37]. One possible explanation for this finding is the carbon imbalance induced by persistent supplementary lighting, which hindered photosynthesis, leaf senescence, and photodamage in tomatoes [38]. Moreover, numerous photoreceptors are involved not only in sensing and responding to light, but also in temperature cues [39]. In previous studies, short days and high temperature accelerated the growth of plants [40]. We found that the effect of the NSL trial in early spring is better than that in the autumn–winter season, which is likely due to the higher temperature in the early spring greenhouse than in the autumn and winter greenhouse. Subsequently, we plan to shorten the duration of night-time supplemental lighting even further to achieve a more effective tomato night-time supplementary lighting pattern.

Using LED to improve the lighting conditions during the process of tomato growth and development is a feasible and efficient method to improve the quality of tomato fruit. Li et al. found that supplementing light in the morning promoted the accumulation of vitamin C, organic acids and sugar [5]. However, we found that the content of soluble sugar, Vitamin C and soluble solids accumulated in tomato fruits subjected to 2 h supplementary illumination was markedly modified compared to that of other treatments in early spring, and the content of titratable acid and Vitamin C in tomato fruits exposed to 4 h night-time lighting supplementation was markedly higher, but the content of soluble sugar in tomato fruits exposed to 5 h night-time lighting supplementation was markedly higher in autumn–winter season. Further research is needed to explain this phenomenon.

## 5. Conclusions

In this experiment, tomato plants were irradiated at night with red and blue dichromatic light at an intensity of 30 μmol·m^−2^·s^−1^, and the results indicated that night-time lighting supplementation can promote plant growth. Therefore, we speculate that night-time supplementary lighting affects tomato production in the form of light signals. Interestingly, the duration of night-time supplemental LED has no effect on tomato yield. The total yield per plant, the yield per trough and the input–output ratio were the highest in the treatments with 1 h of supplemental lighting at night in spring stubble. In order to obtain the maximum economic returns, supplementing lighting for 1 h at night should be selected for tomato cultivation, which has the highest input–output ratio in all supplemental lighting treatments.

## Figures and Tables

**Table 1:** Composition of special nutrient solution for tomato proposed by Yamazaki.

Macronutrients	Final Concentration (mg/L)	Micronutrients	Final Concentration (mg/L)
Ca(NO_3_)_2_·4H_2_O	614	EDTA-FeNa·3H_2_O	6.4
KNO_3_	430	MnSO_4_·H_2_O	1.7
NH_4_H_2_PO_4_	267	ZnSO_4_·7H_2_O	1.5
(NH_4_)_2_SO_4_	33	Na_2_B_4_O_7_·8H_2_O	4.8
MgSO_4_·7H_2_O	430	CuSO_4_·5H_2_O	0.2
K_2_SO_4_	397	Na_2_MoO_4_·2H_2_O	0.2

**Table 2:** Effects of NSL duration on tomato yield and input–output ratio.

NSL Duration (Hours)	The Autumn–Winter Season							
	Single-Fruit Weight (g)	Yield per Plant (kg)	Yield per Trough (kg)	Increased Yield per Trough (kg)	Power Consumption per Trough (kWh)	Power Input per Trough (CNY)	Increased Output (CNY)	Input- Output Ratio
0	120 ± 2.0 b	2.68 ± 0.06 c	104.60 ± 2.50 c	-	-	-	-	-
3	133 ± 0.6 a	3.46 ± 0.09 a	134.94 ± 3.42 a	30.34	63.55	19.07	121.36	1:6.37
4	130 ± 1.0 a	3.21 ± 0.05 ab	125.24 ± 1.91 ab	20.65	85.50	25.65	82.60	1:3.22
5	128 ± 0.9 a	3.13 ± 0.13 b	122.04 ± 5.14 b	17.44	106.35	31.91	69.76	1:2.19
	The early spring season							
0	106 ± 4.0 b	2.45 ± 0.23 b	95.49 ± 5.27 b	-	-	-	-	-
1	114 ± 1.0 ab	2.91 ± 0.04 a	113.68 ± 0.84 a	18.20	24.00	7.20	72.80	1:10.11
2	108 ± 2.0 ab	2.86 ± 0.07 a	111.41 ± 1.52 a	15.93	48.50	14.55	63.72	1:4.38
3	117 ± 2.0 a	2.98 ± 0.03 a	116.05 ± 0.69 a	20.56	70.00	21.00	82.24	1:3.92

**Table 3:** Effects of NSL duration on fruit quality of tomato.

NSL Duration (Hours)	The Autumn–Winter Season					
	Titratable Acidity (%)	Soluble Sugar (%)	Vitamin C (mg/100 g)	Total Soluble Solids (%)	Acid-Sugar Ratio	Total Soluble Solids-Acid Ratio
0	0.25 ± 0.002 b	1.57 ± 0.08 b	16.14 ± 0.84 c	2.93 ± 0.07 c	6.24	11.67
3	0.22 ± 0.002 c	1.64 ± 0.07 ab	19.54 ± 0.34 ab	3.37 ± 0.07 b	7.33	15.00
4	0.27 ± 0.00 a	1.58 ± 0.09 b	21.24 ± 0.87 a	3.03 ± 0.03 c	5.75	11.04
5	0.28 ± 0.006 a	1.87 ± 0.02 a	17.22 ± 0.77 bc	3.97 ± 0.03 a	6.55	13.93
	The early spring season					
0	0.62 ± 0.007 a	1.92 ± 0.04 b	21.54 ± 0.50 b	5.00 ± 0.00 c	3.10	8.12
1	0.54 ± 0.02 b	2.03 ± 0.08 b	21.90 ± 0.49 b	5.17 ± 0.03 b	3.77	9.59
2	0.63 ± 0.006 a	2.49 ± 0.04 a	26.27 ± 0.08 a	6.17 ± 0.03 a	3.94	9.74
3	0.49 ± 0.005 b	1.95 ± 0.009 b	21.56 ± 0.20 b	5.03 ± 0.03 c	3.98	10.27

Different letters indicate statistically significant differences (*p* < 0.05).

## Data Availability

Data are contained within the article.

## References

[1] Li Q., Chai L., Tong N., Yu H., Jiang W. (2022). Potential Carbohydrate Regulation Mechanism Underlying Starvation-Induced Abscission of Tomato Flower. Int. J. Mol. Sci..

[2] Szechyńska-Hebda M., Karpiński S. (2013). Light intensity-dependent retrograde signalling in higher plants. J. Plant Physiol..

[3] Douma J.C., de Vries J., Poelman E.H., Dicke M., Anten N.P., Evers J.B. (2019). Ecological significance of light quality in optimizing plant defence. Plant Cell Environ..

[4] He R., Wei J., Zhang J., Tan X., Li Y., Gao M., Liu H. (2022). Supplemental Blue Light Frequencies Improve Ripening and Nutritional Qualities of Tomato Fruits. Front. Plant Sci..

[5] Wang S., Jin N., Jin L., Xiao X., Hu L., Liu Z., Wu Y., Xie Y., Zhu W., Lyu J. (2022). Response of Tomato Fruit Quality Depends on Period of LED Supplementary Light. Front. Nutr..

[6] Ren M., Liu S., Tang C., Mao G., Gai P., Guo X., Zheng H., Tang Q. (2023). Photomorphogenesis and Photosynthetic Traits Changes in Rice Seedlings Responding to Red and Blue Light. Int. J. Mol. Sci..

[7] de Wit M., Galvão V.C., Fankhauser C. (2016). Light-Mediated Hormonal Regulation of Plant Growth and Development. Annu. Rev. Plant Biol..

[8] Paponov M., Kechasov D., Lacek J., Verheul M.J., Paponov I.A. (2019). Supplemental Light-Emitting Diode Inter-Lighting Increases Tomato Fruit Growth Through Enhanced Photosynthetic Light Use Efficiency and Modulated Root Activity. Front. Plant Sci..

[9] Kucharewicz W., Distelfeld A., Bilger W., Müller M., Munné-Bosch S., Hensel G., Krupinska K. (2017). Acceleration of leaf senescence is slowed down in transgenic barley plants deficient in the DNA/RNA-binding protein WHIRLY1. J. Exp. Bot..

[10] Zhu H., Li X., Zhai W., Liu Y., Gao Q., Liu J., Ren L., Chen H., Zhu Y. (2017). Effects of low light on photosynthetic properties, antioxidant enzyme activity, and anthocyanin accumulation in purple pak-choi (*Brassica campestris* ssp. Chinensis Makino). PLoS ONE.

[11] Gad A.G., Habiba, Zheng X., Miao Y. (2021). Low Light/Darkness as Stressors of Multifactor-Induced Senescence in Rice Plants. Int. J. Mol. Sci..

[12] Monostori I., Heilmann M., Kocsy G., Rakszegi M., Ahres M., Altenbach S.B., Szalai G., Pál M., Toldi D., Simon-Sarkadi L. (2018). LED Lighting—Modification of Growth, Metabolism, Yield and Flour Composition in Wheat by Spectral Quality and Intensity. Front. Plant Sci..

[13] Zou T., Huang C., Wu P., Ge L., Xu Y. (2020). Optimization of Artificial Light for Spinach Growth in Plant Factory Based on Orthogonal Test. Plants.

[14] Samuolienė G., Sirtautas R., Brazaitytė A., Duchovskis P. (2012). LED lighting and seasonality effects antioxidant properties of baby leaf lettuce. Food Chem..

[15] Wang S., Fang H., Xie J., Wu Y., Tang Z., Liu Z., Lv J., Yu J. (2021). Physiological Responses of Cucumber Seedlings to Different Supplemental Light Duration of Red and Blue LED. Front. Plant Sci..

[16] Hamedalla A.M., Ali M.M., Ali W.M., Ahmed M.A.A., Kaseb M.O., Kalaji H.M., Gajc-Wolska J., Yousef A.F. (2022). Increasing the performance of cucumber (*Cucumis sativus* L.) seedlings by LED illumination. Sci. Rep..

[17] Trouwborst G., Oosterkamp J., Hogewoning S.W., Harbinson J., van Ieperen W. (2010). The responses of light interception, photosynthesis and fruit yield of cucumber to LED-lighting within the canopy. Physiol. Plant..

[18] Ntagkas N., de Vos R.C.H., Woltering E.J., Nicole C.C.S., Labrie C., Marcelis L.F.M. (2020). Modulation of the Tomato Fruit Metabolome by LED Light. Metabolites.

[19] Zhang Y., Hu W., Peng X., Sun B., Wang X., Tang H. (2018). Characterization of anthocyanin and proanthocyanidin biosynthesis in two strawberry genotypes during fruit development in response to different light qualities. J. Photochem. Photobiol. B Biol..

[20] Hanssens J., DE Swaef T., Steppe K. (2015). High light decreases xylem contribution to fruit growth in tomato. Plant Cell Environ..

[21] Dannehl D., Schwend T., Veit D., Schmidt U. (2021). Increase of Yield, Lycopene, and Lutein Content in Tomatoes Grown Under Continuous PAR Spectrum LED Lighting. Front. Plant Sci..

[22] Yousef A.F., Ali M.M., Rizwan H.M., Tadda S.A., Kalaji H.M., Yang H., Ahmed M.A.A., Wróbel J., Xu Y., Chen F. (2021). Photosynthetic apparatus performance of tomato seedlings grown under various combinations of LED illumination. PLoS ONE.

[23] Fanasca S., Colla G., Maiani G., Venneria E., Rouphael Y., Azzini E., Saccardo F. (2006). Changes in antioxidant content of tomato fruits in response to cultivar and nutrient solution composition. J. Agric. Food Chem..

[24] Gao Y., Yu H., Liu P., Ma C., Li Q., Jiang W. (2018). Ending composting during the thermophilic phase improves cultivation substrate properties and increasing winter cucumber yield. Waste Manag..

[25] Lazzarin M., Meisenburg M., Meijer D., van Ieperen W., Marcelis L., Kappers I., van der Krol A., van Loon J., Dicke M. (2021). LEDs Make It Resilient: Effects on Plant Growth and Defense. Trends Plant Sci..

[26] Liu P., Li Q., Gao Y., Wang H., Chai L., Yu H., Jiang W. (2019). A New Perspective on the Effect of UV-B on l-Ascorbic Acid Metabolism in Cucumber Seedlings. J. Agric. Food Chem..

[27] Pan T., Wang Y., Wang L., Ding J., Cao Y., Qin G., Yan L., Xi L., Zhang J., Zou Z. (2020). Increased CO_2_ and light intensity regulate growth and leaf gas exchange in tomato. Physiol. Plant..

[28] Steed G., Ramirez D.C., Hannah M.A., Webb A.A.R. (2021). Chronoculture, harnessing the circadian clock to improve crop yield and sustainability. Science.

[29] Borchert R., Renner S.S., Calle Z., Navarrete D., Tye A., Gautier L., Spichiger R., von Hildebrand P. (2005). Photoperiodic induction of synchronous flowering near the Equator. Nature.

[30] Velez-Ramirez A.I., van Ieperen W., Vreugdenhil D., van Poppel P.M.J.A., Heuvelink E., Millenaar F.F. (2014). A single locus confers tolerance to continuous light and allows substantial yield increase in tomato. Nat. Commun..

[31] Tewolde F.T., Lu N., Shiina K., Maruo T., Takagaki M., Kozai T., Yamori W. (2016). Nighttime Supplemental LED Inter-lighting Improves Growth and Yield of Single-Truss Tomatoes by Enhancing Photosynthesis in Both Winter and Summer. Front. Plant Sci..

[32] Zeng Z., Lyu T., Jia X., Chen Y., Lyu Y. (2022). Expression Patterns of Sugar Transporter Genes in the Allocation of Assimilates and Abiotic Stress in Lily. Int. J. Mol. Sci..

[33] Kasperbauer M.J. (1987). Far-Red Light Reflection from Green Leaves and Effects on Phytochrome-Mediated Assimilate Partitioning under Field Conditions. Plant Physiol..

[34] Wallace D.H., Yourstone K.S., Masaya P.N., Zobel R.W. (1993). Photoperiod gene control over partitioning between reproductive and vegetative growth. Theor. Appl. Genet..

[35] Lanoue J., Zheng J., Little C., Grodzinski B., Hao X. (2021). Continuous Light Does Not Compromise Growth and Yield in Mini-Cucumber Greenhouse Production with Supplemental LED Light. Plants.

[36] Nitschke S., Cortleven A., Iven T., Feussner I., Havaux M., Riefler M., Schmülling T. (2016). Circadian Stress Regimes Affect the Circadian Clock and Cause Jasmonic Acid-Dependent Cell Death in Cytokinin-Deficient Arabidopsis Plants. Plant Cell.

[37] Velez-Ramirez A.I., Vreugdenhil D., Millenaar F.F., van Ieperen W. (2019). Phytochrome A Protects Tomato Plants from Injuries Induced by Continuous Light. Front. Plant Sci..

[38] Velez-Ramirez A.I., van Ieperen W., Vreugdenhil D., Millenaar F.F. (2011). Plants under continuous light. Trends Plant Sci..

[39] Ma D., Li X., Guo Y., Chu J., Fang S., Yan C., Noel J.P., Liu H. (2016). Cryptochrome 1 interacts with PIF4 to regulate high temperature-mediated hypocotyl elongation in response to blue light. Proc. Natl. Acad. Sci. USA.

[40] Mao T., Li J., Wen Z., Wu T., Wu C., Sun S., Jiang B., Hou W., Li W., Song Q. (2017). Association mapping of loci controlling genetic and environmental interaction of soybean flowering time under various photo-thermal conditions. BMC Genom..

